# Feasibility of hepatic fine needle aspiration as a minimally invasive sampling method for gene expression quantification of pharmacogenetic targets in dogs

**DOI:** 10.1002/vms3.351

**Published:** 2020-09-20

**Authors:** Matthew B. Hull, Thomas Schermerhorn, Miranda D. Vieson, Jennifer M. Reinhart

**Affiliations:** ^1^ The Department of Veterinary Clinical Medicine College of Veterinary Medicine University of Illinois Urbana IL USA; ^2^ The Department of Small Animal Clinical Science College of Veterinary Medicine Kansas State University Manhattan KS USA; ^3^ Present address: Hill’s Pet Nutrition Topeka KS USA

**Keywords:** drug metabolism, genetics, pharmacology

## Abstract

**Background:**

Quantifying hepatic gene expression is important for many pharmacogenetic studies. However, this usually requires biopsy (BX), which is invasive.

**Objectives:**

The objectives of this study were to determine the feasibility of using minimally invasive fine needle aspirate (FNA) to quantify hepatic gene expression and to assess expression variability between different sampling sites.

**Methods:**

Biopsy and FNA samples were acquired from central and peripheral locations of the right and left lateral liver lobes of a dog. Relative expression of *ABCB1*, *GSTT1* and *CYP3A12* were measured via reverse transcriptase, quantitative PCR. The effect of sampling method, lobe and location within the lobe on gene expression was assessed using a three‐way ANOVA.

**Results:**

Relative expression of *ABCB1* and *GSTT1* were not statistically different between sampling methods but *CYP3A12* expression was higher in samples collected by BX (*p* = .013). Lobe sampled affected *ABCB1* expression (*p* = .001) and site within lobe affected *ABCB1* (*p* = .018) and *GSTT1* (*p* = .025) expression.

**Conclusions:**

FNA appears to be a feasible technique for minimally invasive evaluation of hepatic gene expression but results should not be directly compared to biopsy samples. Sampling location impacts expression of some targets; combination of FNAs from multiple sites may reduce variation.

## Graphical abstract

This study documents that liver samples for gene expression quantification can be obtained minimally invasively via fine needle aspirate rather than invasive biopsy. This technique will allow investigators to much more easily and safely obtain samples for future studies.

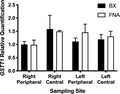

## 1 INTRODUCTION

Variability in hepatic gene expression of xenobiotic‐metabolising enzymes and transporters significantly impacts drug‐related outcomes (Court, 2013). Acquired factors including disease can alter gene expression leading to unexpected therapeutic responses (Ladda & Goralski, 2016; Morgan et al., 2008; Naik, Belic, Zanger, & Rozman, 2013). Genotype also influences hepatic gene expression and clinical outcomes (Court, 2013; Craft, Ekena, Sacco, Luethcke, & Trepanier, 2017; Martinez et al., 2013; Mealey & Fidel, 2015). Thus, quantifying hepatic gene expression is key to linking acquired and genetic factors to drug phenotypes. However, such studies in clinical patients generally requires liver biopsy (BX) to assay gene expression. In dogs, liver BX requires heavy sedation or anaesthesia, carries a risk of bleeding, is invasive and is expensive. Additionally, histopathology from different sites within the same liver can yield different morphologic diagnoses (Kimbrell, Milovancev, Olsen, & Löhr, 2018) and the same could be true for hepatic gene expression.

Fine needle aspiration (FNA) is a minimally invasive technique to sample parenchymal organs including the liver. It can be performed in most dogs with little or no sedation, has a lower risk of bleeding than biopsy, and is inexpensive (Liffman & Courtman, 2017). Furthermore, it is relatively easy to collect and combine samples from multiple areas of an organ, which may allow for a more holistic evaluation of the liver. Fine needle aspiration has not been extensively evaluated as a method to obtain samples for RNA analysis. However, it has been used to detect nucleic acids of pathogens in small animals (Dunbar et al., 2019; Pennisi et al., 2015) and to genotype somatic tumour mutations in humans (Guibert et al., 2018; Jain et al., 2017; Mazeh et al., 2013). Thus, FNA may be useful as a minimally invasive method to quantify hepatic RNA expression of pharmacologically relevant genetic targets in dogs.

The primary objective of this study was to determine the feasibility of FNA to assess hepatic gene expression. A secondary objective was to evaluate variability of expression between different sampling locations within the liver. We hypothesised that neither sampling method (BX versus FNA) nor sampling location would affect hepatic RNA expression of select pharmacogenetic targets.

## 2 MATERIALS AND METHODS

Samples were collected within 30 min of euthanasia from central and peripheral locations of the right and left lateral liver lobes from a 3‐year‐old, female spayed mixed breed dog euthanised for idiopathic renal hematuria and urethral obstruction. Following euthanasia, the animal was donated for research purposes, which is covered under The University of Illinois’s Institutional Animal Care and Use Committee protocol #19137. Wedge BX samples (approximately 200 g each) were divided into four pieces. Three were placed in RNAlater (Thermo Fisher Scientific) for expression analysis. The fourth was examined histologically, which revealed mild changes consistent with systemic inflammation rather than primary hepatopathy with minimal variation between sites (Texts S1–S2). At each site, FNA samples were acquired via fenestration with a 22‐gauge 1.5‐inch needle in triplicate. For each FNA replicate, we performed three fenestrations and passed ~250 μL of RNAlater through the needle (~750 μL total per replicate) to wash the sample into a sterile tube. All samples were stored at 4°C for 24 hr, and then, −80°C.

RNA was extracted using a commercial kit (RNeasy Plus Mini kit, QIAGEN). For BX samples, 20–30 mg tissue was suspended in 600 μL kit lysis buffer and homogenised using a bead‐based tissue disruptor (Digital Disruptor Genie, Scientific Industries Inc). For FNA samples, 2 ml of ice‐cold phosphate‐buffered saline (Thermo Fisher Scientific) was added and samples were centrifuged at 3,300 x g for 10 min. The supernatant was decanted; samples were resuspended in 600 μL kit lysis buffer and lysed via passage through a 22‐gauge needle. All samples were passed through a homogeniser column (QIAshredder, QIAGEN) and RNA quantified using commercial fluorescent assays (Qubit^TM^ RNA XR and HS assay kits, Invitrogen). Biopsy sample quality was assessed using a commercial fluorescent assay (Qubit^TM^ RNA IQ assay kit, Invitrogen) and all IQ numbers were > 8.0. FNA samples contained insufficient RNA quantity for quality assessment (Tables S1 and S2). cDNA was generated from 12 ng RNA using a commercial kit (SuperScript IV VILO, Invitrogen).

We quantified three pharmacogenetic targets: *ABCB1*, *CYP3A12* and *GSTT1*, and two reference genes: *HPRT* and *B2M* (Brinkhof, Spee, Rothuizen, & Penning, 2006). Primer efficiencies were 94.8%–101.3% (Table S3). SYBR Green qPCR with melt curve analysis was performed in triplicate using a commercial kit (QuantiTect SYBR Green RT‐PCR Kit, QIAGEN) on a real‐time PCR system (7,500 Real‐Time PCR System, Applied Biosystems Corp) (Tables S4 and S5).

Statistical analyses for each target were performed separately. For each replicate, expression was normalised to the reference genes and a reaction quotient was calculated using commercial software (Applied Biosystems Relative Quantification Analysis Module, Thermo Fisher Connect, Thermo Fisher Scientific Inc). Data were then normalised to the right peripheral BX sample, since this is the most accessible location via ultrasound. The effect of sampling method (BX versus FNA), lobe sampled (right versus left lateral) and location within the lobe (central versus peripheral) on expression was assessed using a three‐way ANOVA and individual differences identified using Tukey's post hoc test. Statistical analysis was performed using commercial software (Prism 8.0, GraphPad Software) with significance at *p* < .05.

## 3 RESULTS

**FIGURE 1:**
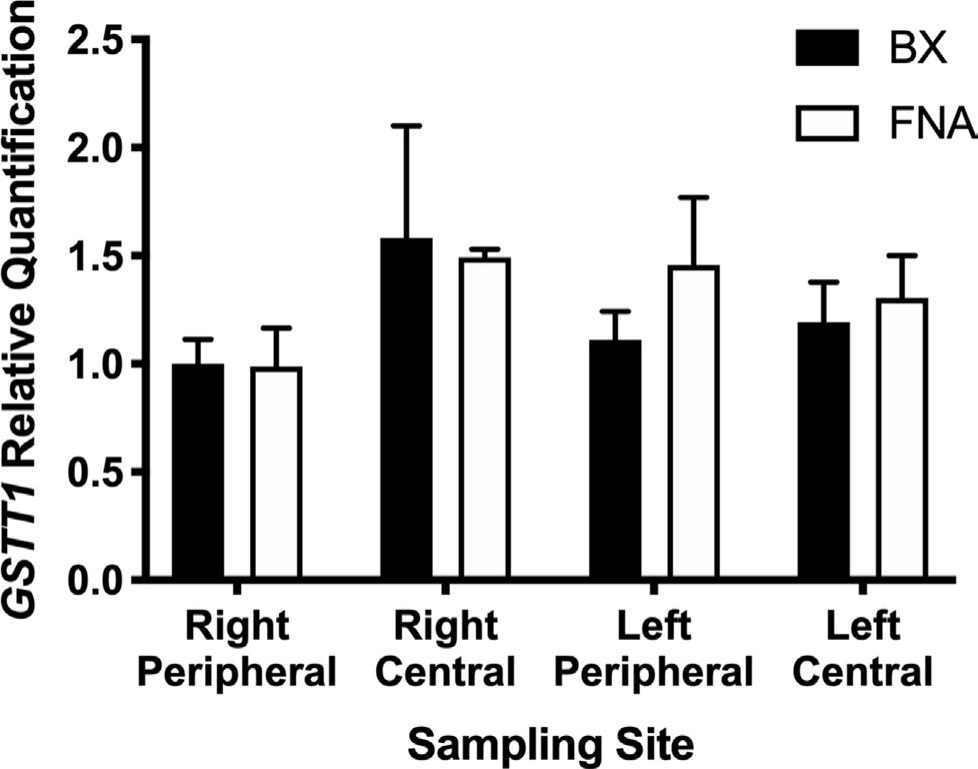
Hepatic expression of *ABCB1* in various liver sites collected by BX versus. FNA. * = significantly different expression compared to left, peripheral BX sample

**FIGURE 2:**
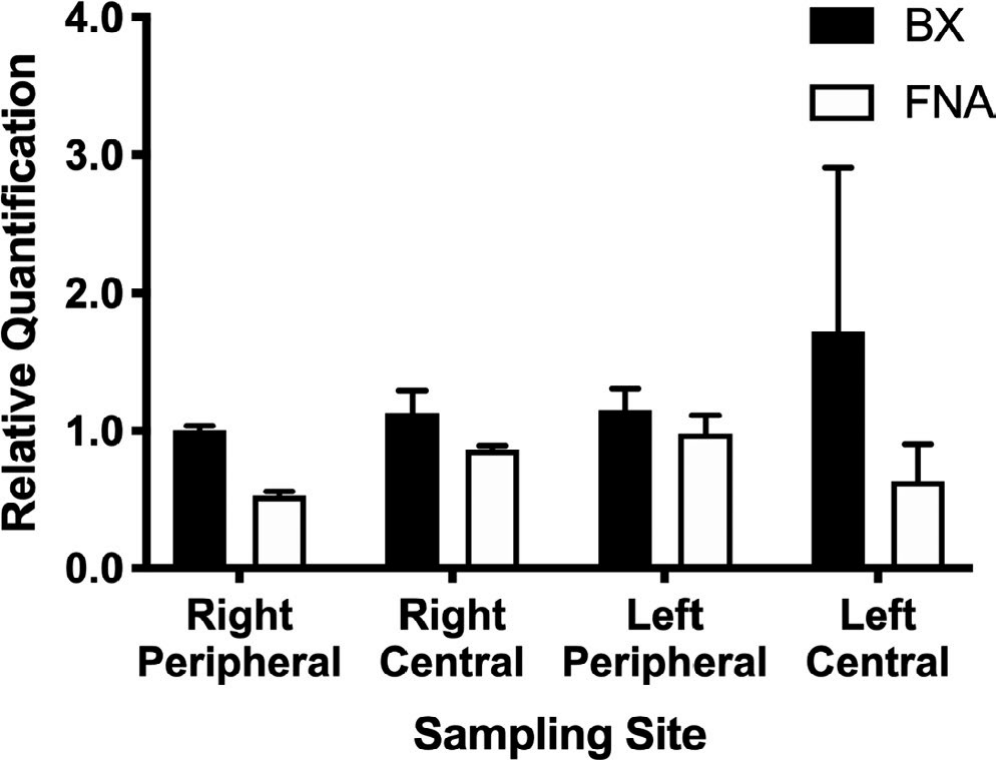
Hepatic expression of *CYP3A12* in various liver sites collected by BX versus. FNA. There were no significant differences between individual samples

**FIGURE 3:**
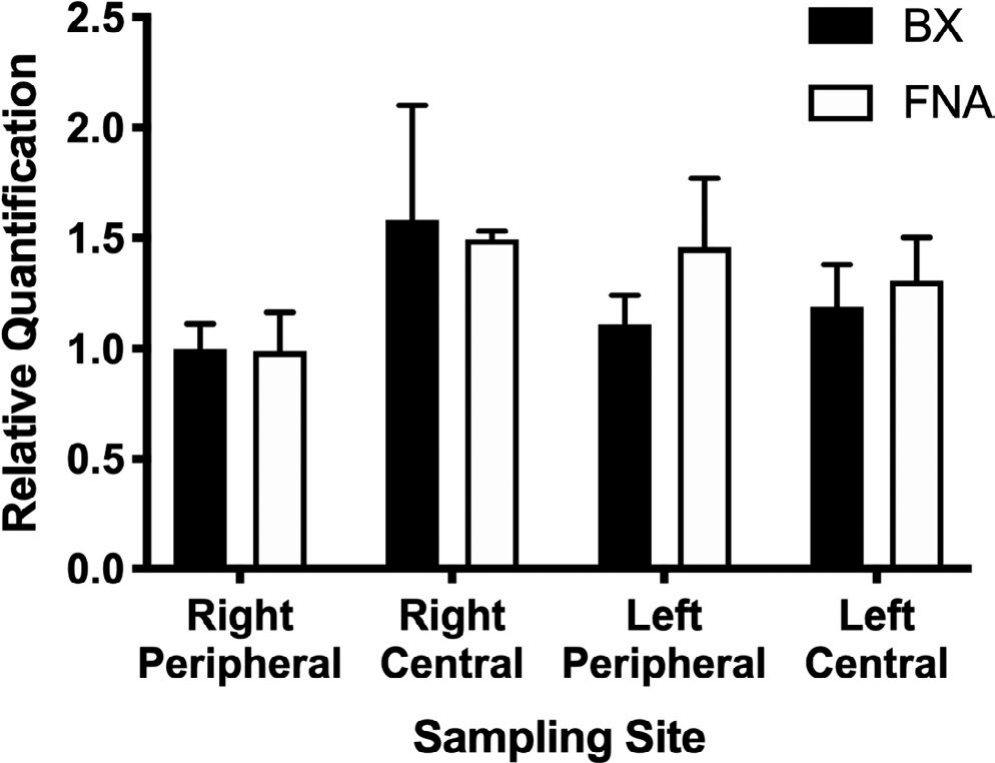
Hepatic expression of *GSTT1* in various liver sites collected by BX versus. FNA. There were no significant differences between individual samples

Expression of all targets was detectable in all samples (Table S6). Relative expressions of *ABCB1*, *CYP3A12* and *GSTT1* are presented in Figures 1, 2 and 3, respectively. ANOVA results for all three targets are presented in Table 1. Briefly, relative expression of *ABCB1* or *GSTT1* was not different between BX and FNA samples but *CYP3A12* expression was higher in samples collected by BX (*p* = .013). Lobe sampled affected *ABCB1* expression (*p* = .001) and site within lobe affected *ABCB1* (*p* = .018) and *GSTT1* (*p* = .025) expression.

**TABLE 1:** Results of three‐way ANOVAs for the effect of the factors sampling method (BX versus. FNA), liver lobe (right versus. left lateral) and location within the lobe (central versus. peripheral) on RNA expression of *ABCB1*, *CYP3A12* and *GSTT1*. * = *p* < .05

Factor	*ABCB1*	*CYP3A12*	*GSTT1*
Sampling Method	0.0871	0.0133*	0.3931
Lobe	0.0009*	0.19833	0.9982
Location	0.0184*	0.3478	0.0246*
Sampling Method x Lobe	0.3065	0.4785	0.1916
Sampling method x Location	0.043*	0.3357	0.4594
Lobe x Location	0.2562	0.7488	0.0123*
Sampling Method x Lobe x Location	0.2974	0.1371	0.7122

## 4 DISCUSSION

Our study demonstrates that RNA expression can be measured in FNA samples from canine liver. Expression levels from FNA samples did not significantly differ from that of BX for two gene targets (*ABCB1*, *GSTT1*) and detectable differences for the third target (*CYP3A12*) were relatively small, on average less than twofold difference between sampling methods. Thus, FNA appears to be a promising technique for minimally invasive assessment of hepatic gene expression in dogs. These findings are similar to that of a recent study in humans, which demonstrated almost identical expression of preselected genes in liver samples acquired by core needle BX versus FNA (Lejnine et al., 2014). The same study confirmed their results in two canine cadaver livers and found similar expression between methods. However, the genes targeted in the canine study were of minimal pharmacologic relevance. Thus, our study is the first to document the feasibility of this technique for genes pertinent to veterinary clinical pharmacology.

Previous histological studies have shown that significant morphologic variability can exist between biopsy samples taken from different sites within the same canine liver (Kimbrell et al., 2018). Therefore, we evaluated multiple liver lobes and locations within lobes to determine whether gene expression would parallel these findings. Expression of 2/3 targets were influenced by the sampling location. Although the differences were small, these findings highlight that variability in expression exists. Thus, it would seem prudent to sample multiple sites within a liver to obtain an average expression level. FNA is amenable to sampling multiple sites because it is an easy and fast procedure with low risk for hemorrhage.

Relative expression results of one target in this study, *CYP3A12*, was significantly different between sampling techniques. Expression at all locations was lower for samples taken by FNA compared to biopsy (Figure 2). These differences were small (less than twofold) for all except one site, the central part of the left lateral lobe. The difference at this location appears to be driven by a single BX replicate, which may be an outlier; however, given our small sample size, we chose not to exclude this datapoint. Because the overall difference between BX and FNA methods was small, this disagreement is unlikely to affect clinical drug outcomes such as efficacy, adverse reactions or even plasma drug levels, since multiple factors influence these phenotypes. However, difference between BX and FNA means that samples collected by different methods should not be used in the same study, which could introduce bias.

Our study has several important limitations. As a feasibility study, our sample size was limited to one dog liver. This precluded evaluation of interindividual variability, which should be assessed in larger, future studies. Additionally, a post‐mortem study allowed specific sampling of different liver lobes, which may not accurately reflect the liver locations accessible by ultrasound in live animals. However, it is unlikely that the location‐related variability documented is specific to the sites sampled. Rather, our results suggest that the specific site sampled is less important than ensuring multiple sites are assessed to account for heterogeneous gene expression. Also, our findings support the use of this technique only for the described target genes in canine liver and results should not be extrapolated to other targets or species without independent validation. The FNA technique used yielded enough RNA for quantification via qPCR but not enough for quality assessment. Thus, differences in RNA quality between FNA and BX samples could have impacted expression results and efforts should be made to improve RNA yield in the future. Finally, our study investigated hepatic gene expression, not expression of protein or enzyme/transporter function, all of which are important when mechanistically linking genotype to clinical phenotype. RNA expression does not always correlate strongly with protein expression for xenobiotic‐metabolising enzymes or transporters and so should not be considered a direct surrogate for protein expression or function (Ohtsuki et al., 2012).

Future studies are needed to further validate the proposed method. Most importantly, the technique should be evaluated in a larger group of animals to assess the degree of interindividual variability in results. The technique should also be evaluated in live animals, to account for ultrasound‐guided accessibility of different sites within the liver for FNA. The effect of histopathologic abnormalities (diffuse and focal) should also be considered for its potential impact on variation in expression between different sites within the same liver. Finally, other FNA techniques should be considered such as aspiration with a syringe in addition to fenestration and larger needle gauges to improve sample yield and allow routine quality assessment of FNA samples.

## 5 CONCLUSION

The genes evaluated in our study represent several classes of gene families that are critical to our understanding of drug disposition, efficacy and adverse reactions (Ginn et al., 2012; Mealey & Fidel, 2015; Perez, Mealey, Grubb, Greene, & Court, 2016). Although hepatic gene expression research has historically been limited by the necessity of surgical biopsies, the findings of our study support FNA as feasible technique for minimally invasive evaluation of hepatic gene expression. Sampling location impacts expression of some targets; combination of FNAs from multiple sites could reduce this variation.

## 6 CONFLICT OF INTEREST STATEMENT

The authors have no conflicts of interest to report.

## 7 ETHICS STATEMENT

The authors confirm that the ethical policies of the journal, as noted on the journal's author guidelines page, have been adhered to. Samples were collected under the University of Illinois Veterinary Teaching Hospital cadaver teaching/research policy and, as such, specific approval of this study by the Institutional Animal Care and Use Committee was not required.

## AUTHOR CONTRIBUTION

**Matthew B. Hull:** Conceptualization; Data curation; Formal analysis; Funding acquisition; Investigation; Methodology; Writing‐original draft; Writing‐review & editing. **Thomas Schermerhorn:** Conceptualization; Formal analysis; Funding acquisition; Investigation; Methodology; Supervision; Writing‐review & editing. **Miranda D. Vieson:** Data curation; Formal analysis; Investigation; Validation; Writing‐review & editing. **Jennifer M. Reinhart:** Conceptualization; Data curation; Formal analysis; Funding acquisition; Investigation; Methodology; Project administration; Software; Supervision; Writing‐review & editing.

### PEER REVIEW

The peer review history for this article is available at https://publons.com/publon/10.1002/vms3.351.

## Supporting information

Supplementary Material
