# Supplementary material for: Feasibility of hepatic fine needle aspiration as a minimally invasive sampling method for gene expression quantification of pharmacogenetic targets in dogs
Source: Vet Med Sci. 2020 Sep 20;7(1):41–5. doi: 10.1002/vms3.351 (PMC7840213; doi:10.1002/vms3.351)
Supplement: Supplementary file 1 — Supplementary Material [file VMS3-7-41-s001.docx]

**Supporting Information**

***Table of Contents:***

Text S1 – Methodology for Histopathologic Evaluation of Liver Samples

Text S2 – Histopathology Report for Liver Samples

Table S1 – RNA Yield and IQ Number for Biopsy Samples

Table S2 – RNA Yield for Fine Needle Aspiration Samples

Table S3 – Primers Used in qPCR Assays

Table S4 – Reaction Mix for qPCR Assays

Table S5 – Cycling Conditions for qPCR Assays

Table S6 – Expression data for *ABCB1*, *CYP3A12*, and *GSTT1*

References

**Text S1** – Methodology for Histopathologic Evaluation of Liver Samples

Liver BXs were fixed in 10% buffered formalin at a 10:1 formalin-to-tissue ratio for at least 24 hours. Formalin-fixed samples were then trimmed into tissue cassettes and underwent routine automated tissue processing through graded alcohols. Samples were then embedded in paraffin, sectioned at 4-5 um, and mounted onto glass slides. Mounted sections were stained with hematoxylin and eosin, cover-slipped, then evaluated by a board-certified veterinary pathologist (MV) with interpretations according to the World Small Animal Veterinary Association guidelines (World Small Animal Veterinary Association Liver Standardization Group, 2006).

**Text S2** – Histopathology Report for Liver Samples

*Liver biopsy results*

Six sections, 2 from each biopsy site (central and peripheral aspects of right lateral and left lateral lobes), are similar and described together. The supportive stroma of central veins is often infiltrated by low numbers of plasma cells, lymphocytes, and neutrophils. Veins with portal triads often contain a mild increase in the numbers of neutrophils and there are rare sinusoids within hepatic lobules that also contain a mild increase in the numbers of neutrophils (presumptive circulating neutrophilia). Approximately 85-95 percent of hepatocytes contain at least one to multiple small (microvesicular), clear, punctate cytoplasmic vacuoles (hydropic degeneration vs lipid) with the peripheral biopsy of the left lateral lobe being most prominently vacuolated, and the peripheral biopsy of the right lateral live lobe being slightly less vacuolated. There are rare (0-2 per examined section), randomly scattered, individual hepatocytes that are hypereosinophilic and contain a karyolytic to karyorrhectic nucleus (single cell necrosis).

*Morphologic Diagnoses:*

1. Mild lymphoplasmacytic perivasculitis with neutrophils

2. Moderate, multifocal to coalescing (random), acute, hepatocellular vacuolar degeneration and rare single-cell hepatocellular necrosis

*Comments:*

Changes in the liver are fairly consistent across all sampling sites with some subtle variations in the degree of hepatocellular vacuolization between the right and left lateral liver lobes. Parenchymal (hepatocellular) changes are acute and reversible characterized by vacuolar degeneration (hydropic vs lipid). There is also rare individual hepatocyte necrosis. These changes are not specific for any particular cause of hepatocellular stress or injury. In this case, it may be related to a systemic inflammatory response as there are slightly increased numbers of neutrophils within veins and sinusoids (presumptive circulating neutrophilia) and a mild non-specific perivasculitis. Another potential cause for a circulating neutrophilia includes an acute stress response (stress leukogram). Overall, a primary hepatitis, cholangitis, cholangiohepatitis, storage disease, biliary obstruction, circulatory anomaly (PSS), and neoplasia are not observed in these sections.

**Table S1 –** RNA Yield and IQ Number for Biopsy Samples. RNA was quantified using the Qubit RNA XR assay and quality was assessed using the Qubit RNA IQ assay. Both assays were performed on the Qubit 4 fluorometer.

| **Sample Type** | **Lobe** | **Location** | **Replicate** | **[RNA] (ng/μL)** | **IQ number** |
| --- | --- | --- | --- | --- | --- |
| BX | Right | Peripheral | 1 | 1,374 | 9.6 |
| BX | Right | Peripheral | 2 | 1,238 | 9.3 |
| BX | Right | Peripheral | 3 | 1,448 | 9.4 |
| BX | Right | Central | 1 | 1,840 | 8.9 |
| BX | Right | Central | 2 | 1,474 | 8.7 |
| BX | Right | Central | 3 | 1,808 | 9.5 |
| BX | Left | Peripheral | 1 | 1,498 | 9.3 |
| BX | Left | Peripheral | 2 | 1,614 | 9.3 |
| BX | Left | Peripheral | 3 | 1,634 | 9.6 |
| BX | Left | Central | 1 | 1,310 | 8.6 |
| BX | Left | Central | 2 | 1,382 | 8.2 |
| BX | Left | Central | 3 | 1,280 | 9.1 |

**Table S2 –** RNA Yield for Fine Needle Aspiration Samples. RNA was quantified using the Qubit RNA HS assay performed on the Qubit 4 fluorometer.

| **Sample Type** | **Lobe** | **Location** | **Replicate** | **[RNA] (ng/μL)** |
| --- | --- | --- | --- | --- |
| FNA | Right | Peripheral | 1 | 0.88 |
| FNA | Right | Peripheral | 2 | 1.23 |
| FNA | Right | Peripheral | 3 | 2.88 |
| FNA | Right | Central | 1 | 2.40 |
| FNA | Right | Central | 2 | 26.80 |
| FNA | Right | Central | 3 | 9.12 |
| FNA | Left | Peripheral | 1 | 42.40 |
| FNA | Left | Peripheral | 2 | 4.08 |
| FNA | Left | Peripheral | 3 | 1.84 |
| FNA | Left | Central | 1 | 45.80 |
| FNA | Left | Central | 2 | 33.20 |
| FNA | Left | Central | 3 | 1.16 |

**Table S3** – Primers used in qPCR assays. *ABCB1*, *CYP3A12*, and *GSTT1* were target genes and *B2M* and *HPRT* were reference genes.

| **Gene** | **Gene Symbol** | **Primers** | **Tm (**^o^**C)** | **Expected Size (bp)** | **Efficiency (%)** |
| --- | --- | --- | --- | --- | --- |
| ATP-binding cassette, subfamily B, member 1 | *ABCB1* | F 5' TGGCCTTATTTTGTGGTTGG 3' | 60 | 109 | 100.244 |
|  |  | R 5' CCTCATCTCGGGTAAAGATCC 3' | 59 |  |  |
| beta-2-microglobulin | *B2M* | F 5' CCTTGCTCCTCATCCTCCT 3' | 59 | 88 | 97.816 |
|  |  | R 5' TCTGCTGGGTGTCGTGAGTA 3' | 60 |  |  |
| cytochrome P-450 3A12 | *CYP3A12* | F 5' CCAGAATTCCAAAGAAATGGA 3' | 59 | 95 | 94.803 |
|  |  | R 5' TGGTCTCATAGCCAGCAAAA 3' | 59 |  |  |
| glutathione-S-transferase theta 1 | *GSTT1* | F 5' TGCCCGTGTGGATGAGTA 3' | 59 | 98 | 101.91 |
|  |  | R 5' CCAGGAAAACAGGGAACATC 3' | 59 |  |  |
| hypoxanthine guanine phosphoribosyl transferase | *HPRT* | F 5' TGGAAAGAATGTCTTGATTGTTG 3' | 59 | 88 | 101.281 |
|  |  | R 5' TGGATTATGCTCCTTGACCA 3' | 59 |  |  |

**Table S4** – Final contents of each SYBR Green qPCR reaction. Each sample/control was performed in triplicate.

| **Reagent** | **Volume** |
| --- | --- |
| SYBR Green 2x Master Mix | 12.5 μL |
| Primers (Forward 10 μM, Reverse 10 μM) | 0.8 uL |
| cDNA or no-reverse-transcriptase control (1:10) | 6.2 μL |
| Nuclease-free water | 5.5 μL |
| TOTAL | 25 μL |

**Table S5** – Cycling Conditions for qPCR Assays. Assays were performed on an Applied Biosystems 7500 RealTime PCR system.

| **Step** | **Time** | **Temperature** | **# of Cycles** |
| --- | --- | --- | --- |
| Initial Activation | 15 min | 95^o^C | 1 |
| Denaturation | 15 sec | 94 ^o^C | 45 |
| Annealing | 30 sec | 55 ^o^C |  |
| Extension and  Data Acquisition | 30 sec | 72^o^C |  |

**Table S6 –** Reaction quotients for each sample replicate. Reaction quotients were normalized such that the mean reaction quotient for the right peripheral BX sample was 1.0, since this is the location most likely to be sampled via FNA clinically.

| **Sample Type** | **Lobe** | **Location** | **Replicate** | **ABCB1** | **CYP3A12** | **GSTT1** |
| --- | --- | --- | --- | --- | --- | --- |
| BX | Right | Peripheral | 1 | 0.8517 | 1.0386 | 1.0952 |
| BX | Right | Peripheral | 2 | 1.0865 | 0.9677 | 1.0303 |
| BX | Right | Peripheral | 3 | 1.0617 | 0.9937 | 0.8745 |
| FNA | Right | Peripheral | 1 | 0.6384 | 0.4933 | 0.9293 |
| FNA | Right | Peripheral | 2 | 0.6483 | 0.5442 | 1.1876 |
| FNA | Right | Peripheral | 3 | 0.5838 | 0.5465 | 0.8514 |
| BX | Right | Central | 1 | 0.5276 | 1.2776 | 1.6825 |
| BX | Right | Central | 2 | 0.6830 | 0.9535 | 1.0202 |
| BX | Right | Central | 3 | 0.4697 | 1.1569 | 2.0447 |
| FNA | Right | Central | 1 | 0.5689 | 0.8506 | 1.4517 |
| FNA | Right | Central | 2 | 1.0717 | 0.8435 | 1.5195 |
| FNA | Right | Central | 3 | 0.7409 | 0.8943 | 1.5094 |
| BX | Left | Peripheral | 1 | 1.5910 | 1.3237 | 1.2583 |
| BX | Left | Peripheral | 2 | 1.2635 | 1.0304 | 1.0693 |
| BX | Left | Peripheral | 3 | 1.6009 | 1.0919 | 1.0043 |
| FNA | Left | Peripheral | 1 | 1.3991 | 1.1309 | 1.8182 |
| FNA | Left | Peripheral | 2 | 0.8550 | 0.9263 | 1.2886 |
| FNA | Left | Peripheral | 3 | 1.0832 | 0.8766 | 1.2626 |
| BX | Left | Central | 1 | 0.7178 | 1.0753 | 1.0534 |
| BX | Left | Central | 2 | 0.8120 | 3.0899 | 1.1183 |
| BX | Left | Central | 3 | 1.5496 | 1.0032 | 1.4040 |
| FNA | Left | Central | 1 | 0.9013 | 0.9014 | 1.3593 |
| FNA | Left | Central | 2 | 0.8765 | 0.3691 | 1.0880 |
| FNA | Left | Central | 3 | 0.8104 | 0.6317 | 1.4690 |

**References**

World Small Animal Veterinary Association Liver Standardization Group, Rothuizen, J., Bunch, S. E., Charles, J. A., Cullen, J. M., Desmet, V. J., Szatmari, V., Twedt D. C., van den Ingh, T. S. G. A. M., Van Winkle T., Washabau, R. J. (2006). *WSAVA standards for clinical and histological diagnosis of canine and feline liver diseases*. Saunders Ltd.
